# Applying Normalisation Process Theory to a peer-delivered complex health intervention for people experiencing homelessness and problem substance use

**DOI:** 10.1038/s43856-024-00721-6

**Published:** 2025-01-10

**Authors:** Rebecca Foster, Hannah Carver, Catriona Matheson, Bernie Pauly, Jason Wallace, Graeme MacLennan, John Budd, Tessa Parkes

**Affiliations:** 1 School of Applied Sciences, Sighthill Campus, Edinburgh Napier University, Edinburgh, Scotland UK; 2 Salvation Army Centre for Addiction Services and Research, Faculty of Social Sciences, Colin Bell Building, University of Stirling, Stirling, Scotland UK; 3 Nursing, Midwifery and Allied Health Professions Research Unit, Centre for Healthcare and Community Research, University of Stirling, Stirling, Scotland UK; 4 Canadian Institute for Substance Use Research, University of Victoria, Victoria, Canada; 5 Scottish Drugs Forum, 91 Mitchell Street, Glasgow, Scotland UK; 6 Centre for Healthcare Randomised Trials, Health Sciences Building University of Aberdeen, Foresterhill, Aberdeen, Scotland UK; 7 University of Edinburgh Medical School, Chancellor’s Building, 49 Little France Crescent, University of Edinburgh, Edinburgh, Scotland UK

**Keywords:** Public health, Clinical trial design

## Abstract

**Background:**

The Supporting Harm Reduction through Peer Support (SHARPS) study involved designing and implementing a peer-delivered, harm reduction intervention for people experiencing homelessness and problem substance use. Normalisation Process Theory (NPT) provided a framework for the study.

**Methods:**

Four Peer Navigators (individuals with personal experience of problem substance use and/or homelessness) were recruited and hosted in six third sector (not-for-profit) homelessness services in Scotland and England (United Kingdom). Each worked with participants to provide practical and emotional support, with the aim of reducing harms, and improving well-being, social functioning and quality of life. NPT guided the development of the intervention and, the process evaluation, which assessed the acceptability and feasibility of the intervention for this cohort who experience distinct, and often unmet, health challenges. While mixed-methods data collection was undertaken, this paper draws only on the qualitative data.

**Results:**

The study found that, overall, the intervention is feasible, and acceptable to, the intervention participants, the Peer Navigators and staff in host settings. Some challenges were encountered but these were outweighed by benefits. NPT is particularly useful in encouraging our team to focus on the relationship between different aspects of the intervention and context(s) and identify ways of maximising ‘fit’.

**Conclusions:**

To our knowledge, this is the first application of NPT to this cohort, and specifically by non-clinicians (peers) in non-healthcare settings (homelessness services). Our application of NPT helped us to identify ways in which the intervention could be enhanced, with the key aim of improving the health/well-being of this underserved group.

## Plain language summary

This paper shares findings from a study which aimed to improve the health and well-being of people experiencing problem substance use (alcohol/drugs) and homelessness. The intervention involved peers, people with first-hand experience of these challenges, delivering an intervention that was person-centred, trauma informed and based on harm reduction principles. To help us identify if the intervention was practical and had value, we conducted a process evaluation guided by a framework called Normalisation Process Theory (NPT). In using NPT, we were able to pinpoint areas that needed to be improved, and sometimes we were able to make changes at the time. We will use this learning going forward to design an intervention that best meets the needs of this overlooked group.

## Abstract (web summary)

Foster, Carver et al. apply Normalisation Process Theory (NPT) to provide an understanding of how successful the Supporting Harm Reduction through Peer Support study (SHARPS) was. Overall the intervention was feasible, and acceptable to, the intervention participants, the Peer Navigators and staff in host settings, with challenges outweighed by benefits.

## Introduction

People who report being homeless often experience co-occurring poor mental health, poor physical health, and problem substance use (drugs and/or alcohol), also termed ‘substance use disorders’, alongside a range of other compounding challenges^1,2^.The health impacts of homelessness are severe and long-lasting^3–5^. Accessing services, including healthcare, can be burdensome; for example, individuals can encounter stigmatising attitudes from staff, inflexible processes, and disjointed systems^6,7^. Research has highlighted the importance of relationships with trusting, non-judgemental service staff^8^. Peer-delivered approaches seem to have promise in helping others and have been shown to be beneficial to the peers themselves. The benefits to those whom peers are supporting include developing trust and connections, reducing substance use related harms, and improving housing outcomes; and the benefits to the peers themselves range from improving their own lives, having a sense of purpose, reducing substance use, and career development^6,9–12^. While different definitions are used, the nuance of which we recognise, peers are generally considered to be individuals with lived, or first-hand, experience of a particular issue or challenge who use that experience in their work^12^. Peer support is found in a range of contexts and has been increasingly applied in the substance use and homelessness fields^12^. Peer support can be informal/ad hoc; alternatively, it can be formalised with recognised, designated roles in either a voluntary or paid capacity^12^. Peer-delivered approaches involve formalised peer support where peers are trained to deliver an intervention or service to individuals experiencing similar challenges^13^. Prior to the SHARPS study, there was a lack of research evidence on the acceptability and effectiveness of peer-delivered interventions specifically for individuals experiencing homelessness and problem substance use^6^.

The SHARPS study drew on academic and grey literature evidence on harm reduction, peer-delivered interventions, and Psychologically Informed Environments (PIEs) to develop and implement a unique, complex health intervention. The aim of the intervention was to reduce harms and improve health/well-being, quality of life and social functioning for individuals experiencing homelessness and problem substance use. The intervention was delivered by peers: people with their own, diverse lived experience of homelessness and/or problem substance use. The intervention was delivered to a cohort of people dually experiencing homelessness and problem substance use, across six intervention sites, all of which were managed by third sector (not-for-profit) services in Scotland and England in the United Kingdom (UK). The peers, termed ‘Peer Navigators’, worked closely with individuals to address a range of health/social issues, through providing practical and emotional support. SHARPS was a two-year (May 2018–May 2020) mixed-methods feasibility study whose overarching aims were to design and implement the ‘Peer Navigator’ intervention, and to conduct a concurrent process evaluation. The feasibility and acceptability of procedures were tested to inform a potential randomised controlled trial (RCT). Full details of the SHARPS study, including how the intervention was developed, can be found in related publications^6,14^.

Since its development between 1998 and 2008^15^ Normalisation Process Theory (NPT), has been used to inform feasibility studies and process evaluations for a wide, and growing, range of complex healthcare interventions^16,17^ with generally positive responses from researchers and practitioners alike^17,18^. As Murray et al.^19^ outline, NPT recognises that healthcare is collective and requires a range of interactions from different actors. It provides a clear framework to help understand the effects of these interactions: for instance, in how these can help, or hinder, the implementation of an intervention. Nonetheless, NPT is also intended to be flexible^20^ which is perhaps evidenced by its extensive application. NPT has a role in the development, implementation, and evaluation of complex interventions^17,19^: in other words, NPT can be applied from intervention inception, through to post-implementation review. NPT proposes that implementation occurs through ‘four generative mechanisms’^21,22^. These four mechanisms are typically referred to as its main constructs: coherence, cognitive participation, collective action, and reflexive monitoring^21,22^. Coherence relates to the cognitive process individuals/organisations undergo to either support or impede an intervention from being implemented (‘understanding’); cognitive participation involves individuals/organisations engaging in the new intervention (‘buy-in’); collective action refers to the activity that individuals/organisations do to put the intervention into practice (‘making it work’); and reflexive monitoring concerns the informal and formal assessment that individuals carry out to consider how the intervention affects them/their organisation (‘on-going appraisal’)^14^. These core constructs contain four sub-components^21,22^. The constructs are not linear and do not exist independently of one another; rather, they are inter-connected^19^. As a ‘middle-range theory’, NPT can be applied to a range of methodologies and is well-placed to support the qualitative components of process evaluations of complex interventions, including to support qualitative data analysis^22^.

Huddlestone et al.^20^ recommend that researchers provide explanations for their selection of NPT to aid decision-making for others. In response to this, SHARPS was complex intervention in line with Medical Research Council and National Institute for Health and Care Research definitions^23,24^, making NPT a good fit. Interventions are considered complex if they involve multiple components; require specific skills/expertise; have a large number of groups or settings; require flexibility; or target a range of behaviours^23,24^. Process evaluations are key to understanding how such complex interventions work, including the context, mechanisms and the implementation process^25^.

During intervention development, we considered how we could maximise the ‘fit’ of the intervention across all settings. For example, we drew on the expertise of practitioners, clinicians, and individuals with lived experience, to identify potential facilitators and pitfalls^6^. We concur with others who have commented that there is scope to use NPT more *prospectively*, in these important planning stages^18–20^. While we used NPT to guide the intervention design and development, we primarily used NPT in our process evaluation and within this, we found it to be particularly useful in guiding qualitative data analysis. In light of this, and also considering space constraints, this paper focuses on this aspect. Therefore, for clarity, this paper specifically focuses on how we used NPT in the qualitative component of our process evaluation to assess feasibility and acceptability, foregrounding the experiences of those directly involved in the intervention; other aspects relating to feasibility are presented in related publications^6,14^.

Overall, the SHARPS study was viewed to be feasible for, and accessible and acceptable to intervention participants, Peer Navigators and service staff. Using NPT we have identified a range of implementation benefits and challenges, including how the intervention was received and how the Peer Navigators were able to deliver the intervention. More detail on the wider study findings can be found in in our associated publications^6,14^.

## Methods

### The SHARPS study

To give essential context to our discussion of how we applied NPT, we now provide a brief overview of the study and intervention. Full ethical approval for the study was sought and obtained. The University of Stirling’s NHS, Invasive and Clinical Research (NICR) ethics committee (NICR 17/18 Paper 2018) provided ethical approval for this study in April 2018, and The Ethics Subgroup of the Research Coordinating Council of The Salvation Army (TSA) in June 2018 (no reference number provided). In response to necessary protocol amendments, four subsequent submissions to these committees were made, all of which were approved. Study participants gave their consent for the publication of research data, as part of the informed consent process.

As mentioned, the SHARPS study involved designing and implementing a peer-delivered, harm reduction-based intervention, informed by the principles of PIEs. Briefly, harm reduction is an approach that aims to reduce the harms associated with substance use and promote safer use. It aims to respond to individual needs at a given time, rather than encouraging change before they may be ready or able to do so^26^. For example, abstinence may not be possible at a particular juncture, but responding to the harms of substance use/substance use disorder can be an achievable goal. Given the challenges outlined, as well as experiences of previous/current trauma that are common to those experiencing homelessness^27,28^. Homelessness settings (both accommodation and outreach) in the UK are becoming more trauma sensitive^14^. Many services now incorporate the principles of PIEs. The PIEs approach offers a way of understanding how individual’s responses to situations, including their thoughts, feelings, and actions, can be influenced by their past and present experiences^29^. Services which adopt a PIEs approach prioritise the staff-client relationship, including staff responses to manifestations of trauma; for example, regular staff reflective practice sessions are a central feature of PIEs^30,31^.

### The SHARPS intervention

The study was commissioned by the NIHR in 2017. The Peer Navigator role was developed by members of the SHARPS team with academic and practitioner experience to be a role similar to that of a support worker but with the added benefits of: lived experience; being able to go with people to attend appointments or services; having a practical support fund to buy small items; and being able to provide support even if an individual left a service. Unlike some of the staff members in the host services (e.g., residential support workers), the Peer Navigators were not required to be desk-based. While the role was quite unique within the involved services, it is similar to those of other roles in the field. Four Peer Navigators were recruited and employed by The Salvation Army (TSA), for 18 months and worked 30 h per week; one Peer Navigator left the role early for a number of personal and professional reasons including the travel required to perform the role. During the 3.5-month induction period, the Peer Navigators received extensive training on a range of relevant areas (such as trauma-informed care, motivational interviewing, and naloxone administration). Taking account of the need to provide varied and comprehensive support, the Peer Navigators benefitted from both informal (e.g., mentorship) and formal (e.g., monthly clinical supervision) support to help them carry out their roles, including from the study team. They were hosted in three outreach services for people experiencing homelessness in Scotland, operated by different providers (namely, the TSA; Streetwork/Simon Community Scotland; and Cyrenians/Change Grow Live), and three TSA hostels in England. Two of the Peer Navigators worked across a TSA drop-in service and two other services, another worked in one TSA hostel, and the fourth worked across two TSA hostels within the same city. Those working across multiple services/service providers split their time equally between the sites, ensuring sufficient time to become integrated into the settings and to provide support to their caseload. Service contexts varied, including in terms of the extent to which they embraced harm reduction.

The Peer Navigators worked with a caseload of around 15 participants each (total intervention participants *n* = 68), for a period of 2–12 months. Individuals were eligible to take part if they were over 18 years old; were experiencing homelessness or at risk of homelessness (often chronic); self-identified as having a problem with alcohol and/or drugs that they considered to be negatively affecting their lives; and were able to give informed consent. Given the person-centred orientation of the intervention, the support provided was highly individualised and changed over the course of the intervention. To give an indication of the breadth of support offered, the Peer Navigators helped participants to find appliances for their new tenancies (after moving out of hostels); supported participants when arranging and attending a range of healthcare appointments including those relating to pregnancy, substance use, nutrition, mental health, and post-operative care; and provided a listening ear to participants experiencing relationship breakdown or challenges maintaining family contact. More details of the support provided can be found in our associated publications^6,14^.

### Mixed-methods methodology

As part of a larger mixed methods study, qualitative data were collected from various sources and at different time points. Semi-structured interviews were undertaken with a sample of intervention participants at two time points (*n* = 24, and *n* = 10), to capture any changes in their views about the intervention over time. The interviews were not re-attempted in the site where there was the shortened intervention due to the Peer Navigator leaving the role. The other reasons for not being able to re-interview all participants from the first time point were varied and included the participant being in custody, being physically or mentally unwell, or securing employment incompatible with scheduled interview times. Interviews were conducted with staff working in the intervention settings (*n* = 12), and with the Peer Navigators at four time points; three for the Peer Navigator who left the role early. Interview schedules are provided as Supplementary File 1. Academic researchers (RF, HC) from the study team conducted the staff and Peer Navigator interviews, and peer researchers (*n* = 8) from the Scottish Drugs Forum conducted the intervention participant interviews. The peer researchers were volunteers with lived/living experience of problem substance use and trained in research methods. Academic researchers also conducted observations in all settings, and the Peer Navigators kept reflective diaries. Quantitative data were collected via six standardised measures, broadly on demographic characteristics, problem substance use, physical and mental health, and housing circumstances. These data provided useful insights about the unique circumstances of the cohort, and also demonstrated that the participants were able to remain engaged with the intervention, despite experiencing a host of challenges; quantitative findings are shared in other publications^6,14^. This paper solely reports the qualitative data collected.

### Qualitative data analysis and NPT as guiding framework

All interviews were transcribed verbatim and analysed using Framework Analysis^32^, a popular tool in qualitative health research^33^, and aided by NVivo (Version 12)^34^ computer qualitative data analysis software package. Framework Analysis enabled analysis of data from all six settings and between/within case comparisons. NPT was used as a ‘guiding framework’ to help identify the contextual factors which impacted implementation, and ultimately to assist the assessment of the acceptability and feasibility of SHARPS, as per our research aims. Data analysis was iterative and led by academic researchers. In addition, our Experts by Experience group members (Patient and Public Involvement group), and a sample of the peer researchers participated in data analysis/interpretation sessions, led by RF and HC. We hoped that this type of ‘member checking’^35^ would enhance the rigour of our findings, for example, highlighting themes perhaps more readily identified by those with first-hand experience of the issues raised in interviews, such as prior experience of ‘feeling unheard’ when interacting with services. Further detail on this process from the perspective of our Experts by Experience group is provided in our related paper^36^.

### Reporting summary

Further information on research design is available in the Nature Portfolio Reporting Summary linked to this article.

## Results and discussion

We now discuss each of the four core concepts with reference to specific examples from our qualitative data: specifically, interviews with intervention participants, Peer Navigators, and service staff. While we have categorised our analysis and illustrative quotations into these discrete concepts, we recognise their inter-relatedness and the overlaps. To avoid identifying the individual Peer Navigators, we have changed gender pronouns to ‘they’, and we denote them as ‘Peer Navigators A-D’. Usage of the Scots dialect/language has been retained, with translations provided, where applicable.

### Coherence (‘understanding’): “they took me on to help”

Coherence relates to how individuals make sense of a new intervention. Through observing and interacting with the Peer Navigators in the host services, intervention participants were quick to understand what the Peer Navigator intervention involved, and to identify its potential to support them. For some, this ultimately led to their decision to take part.

*“I remember seeing them over there the first few times and they told me what it [the study] was all about […]. We started chatting and I asked ‘could you be my Support Worker?’ because I seen [saw] them helping out other people.”* (Intervention participant)

*“In here [the service], they were talking to someone, but they were always dead friendly and obviously they took me on to help.”* (Intervention participant)

Senior leaders and service managers within the host organisations received a comprehensive introduction to the SHARPS intervention by the study team. While the aim was for this information to have been fully communicated to all frontline staff, this was not always done fully, or able to have been done (for example, due to service pressures). Consequently, some service staff took longer to understand the intricacies of the intervention, and how it could fit within their workplace. Some staff and managers talked about challenges arising between the Peer Navigators and other staff members in services.

*“Initially there was a bit of suspicion around, around the post, and not really having an idea of what it was and I think on reflection if I was to go back I’d maybe take more responsibility and explaining more what the role was, I think that might have been more beneficial.” (Staff participant)*

Peer Navigators also recognised similar issues including role confusion among some service staff, and the tensions which sometimes manifested as a result.

*“[…] we did clash quite a few times because they didn’t understand my role.” (Peer Navigator)*

These tensions will be discussed again shortly. Despite some challenges with ‘coherence’, in general, staff understood the benefits offered by the Peer Navigators’ lived experience and the flexible, person-centred role.

*“It’s been great for us. Once [clients] move on we don’t get to see them, so it was great to see the progression of one of my lads that [Peer Navigator A] was looking after, and how he was getting along. They were able to go to the viewing of the flat with him. I think as a team member they fitted in well because they had those freedoms that we would love to do.” (Staff participant)*

The Peer Navigators were very receptive to the ethos of the intervention, and quickly developed a clear understanding of the intervention and their role in it.

*“As Peer Navigators, the main thing that we’ve got on our side is time […] it’s very much ‘we are in this together’ kind of relationship. We will do this. We will go and do that.” (Peer Navigator)*

Overall, the Peer Navigator intervention was well understood by participants, albeit with some confusion over the role, especially at the start. Staff understood the benefits of the role and the importance of the Peer Navigators’ lived experience.

### Cognitive participation (‘buy-in’): “really valuable”

Cognitive participation relates to individuals ‘buying into’ the new intervention. Linking with the discussion on ‘coherence’, staff members tended to believe that the Peer Navigators brought a range of benefits to the team and the service, which helped them to ‘buy into’ the intervention. This created opportunities for different forms of engagement, including outreach work.

*“So that type of stuff [outreach work] has been really valuable. It’s that ability to respond really quickly, whereas if you are in a staffed centre, the staff can’t leave the centre to go elsewhere.” (Staff participant)*

The intervention was also felt to provide strategic benefits, raising the organisation’s profile in the not-for-profit sector for its involvement with this innovative intervention:

*“It’s raised the profile of the organisation because [the Peer Navigators] have been able to show that the organisation, which has been about for such a long time, is trying new things, it’s doing research.” (Staff participant)*

The identification of these benefits cemented support of the intervention among some staff members. However, as this was a feasibility study, staff participants were encouraged to consider the intervention’s application on a wider scale, both in terms of size and fit beyond the third sector (e.g., healthcare settings, specifically the NHS in the UK context of the study). For some staff, there was a cautious ‘buy-in’, where staff were supportive of the SHARPS intervention, but identified potential challenges for wider roll-out. For example, staff reflected on the lengthy (3.5-month) induction period and the extensive training the Peer Navigators received, and questioned whether this would be a realistic prospect if rolled out.

*“That’s a funny thing when you are used to people turning up and just getting on with the job. I’ve got two new workers who started on Monday and the expectation is that by Thursday they will be doing some of the tasks.” (Staff participant)*

*“All the money for the training was great, but I don’t know how realistic that would be with other organisations, or if it was training massively outwith [beyond] [the local area].” (Staff participant)*

In summary, there was generally good ‘buy-in’ to the intervention among intervention participants, staff and the hosting organisations. However, some staff members, familiar with the constraints of the third sector (especially resource pressures), identified potential challenges for roll-out.

### Collective action (‘making it work’): “I just made space for them, you know”

Collective action relates to the work individuals do to embed an intervention in a setting. While the Peer Navigator intervention was generally positively received by staff, some tensions did manifest, meaning more ‘work’ was needed to embed the intervention. For example, one staff member described how they tried to accommodate the Peer Navigator within their staff team:

*“I squashed that resistance. I just made the space for them, you know, and they could then get on. And I think there was a degree of acceptance about that from the members of staff. But […] it’s absolutely nothing to do with your project and it’s nothing to do with [Peer Navigator B’s] personality, it’s limitations within my staff team.” (Staff participant)*

The Peer Navigators appeared to be highly attuned to the feelings and perceptions of some of members of staff. For example, identifying that the novelty of the role (at least within the services involved), and the privileges it offered, such as flexibility and freedom, could be confronting or threatening to existing staff in these services.

*“Part of it might be that [staff] see it as a threat, or they see it as a new-fangled thing, a new fad.” (Staff participant)*

To help address these tensions, and ultimately, to help the intervention ‘fit’, the Peer Navigators also engaged in their own work (‘action’):

*“This is what I’ve tried to explain to the staff, sit down and talk to them sometimes. And I’ve said ‘listen, my role is different from the support work that you are giving’. So, it’s like, ‘there is nothing wrong with us all having different methods and different approaches about how we interact with people.’” (Peer Navigator)*

Connecting with the discussion of ‘coherence’, part of the implementation challenges related to it simply taking time for all actors to understand and buy in to the intervention, and for them to be willing to make the necessary adjustments to make it work. Reflecting, one staff participant commented *“I think that this* [the intervention] *has worked pretty well”* but prefaced this with*:* “*I felt like it was a bit sticky at the beginning”*.

Overall, there was a positive view of the Peer Navigator intervention, although some participants described a lack of clarity over the role and tensions between the Peer Navigators and members of the existing staff team. There was a sense that the Peer Navigator role could be a threat to other staff, requiring the Peer Navigators to try to alleviate these tensions and for the role to have been explained more fully at the beginning by management. Over time, the intervention was generally better accepted.

### Reflexive monitoring (‘on-going appraisal’): “I do think through my own process”

Reflexive monitoring concerns how individuals appraise the intervention, including its impacts on themselves and others. As mentioned, the Peer Navigators had first-hand experience of problem substance use and/or homelessness, but the nature of these experiences was, inevitably, highly varied. Each Peer Navigator had their own routes out of the challenges they faced, and a distinct approach to their personal recovery. For some, this jarred with the explicitly harm reduction focus of the SHARPS intervention, at least in the beginning, as they reflected.

*“I do think through my own process, from when I first started, that I’ve arrived at a much more harm reduction-focused approach. And my attitude towards harm reduction, and what harm reduction is and what harm reduction isn’t, has changed a lot.” (Peer Navigator)*

*“I saw a very sharp divide between recovery and harm reduction. I would love to be involved now in the sort of harm reduction stuff […] and that’s not what I anticipated would be one of the outcomes.” (Peer Navigator)*

While not about the Peer Navigators themselves, it is important to note the wider service context where harm reduction was very well accepted in some of the services where they were based but less so in others. Some services had staff members who were not supportive of harm reduction approaches, whereas the Peer Navigators were working within a harm reduction ethos. This was not linked to the Peer Navigator who left the project early, however. Linking with earlier discussion, one staff member reflected that if the frontline staff within *services* had been engaged with at an earlier stage, implementation could have been smoother.

*“That is what we’ve learned. Staff would have felt perhaps more informed and been able to say right at the beginning ‘how would that work?’, and we could have thought about it a bit more and thought about it together.” (Staff participant)*

While research teams may decide to take reflexive notes, for a range of reasons, such as for accountability purposes, NPT encourages active, on-going reflexive monitoring through this component, meaning that this is built into the process evaluation. In response, our team took detailed notes throughout the duration of the study. These notes encompassed personal views and feelings, alongside notes from conversations, meetings, and interactions. We used these to help contextualise the data, to determine acceptability and feasibility^6^. However, reflexive monitoring is also about continuous evaluation^21^. Therefore, being attentive to the Peer Navigators as they delivered the intervention across all settings, was essential not only for their well-being, but to optimise the intervention. For example, there were points when the study team’s informal support of the Peer Navigators needed to be more dynamic or intensive. For instance, a member of the study team was available for ad hoc calls or texts: to be a ‘sounding board’ for ideas for supporting participants (“did I do the right thing there?”), or to listen to frustrations about (what was perceived to be) bureaucratic processes and challenges with partnership working.

At the beginning, some of the Peer Navigators struggled with the harm reduction ethos due to their own recovery journeys, but overtime their viewpoint changed. Often, this was related to greater exposure to harm reduction in practice in their host services, coupled with an appreciation that, for some, harm reduction was a more achievable goal than abstinence. Staff felt that engagement early in the intervention would have ensured a smoother journey throughout the intervention. Provision of informal and formal support to the Peer Navigators ensured continuous reflection on the intervention, their roles, and the challenges they faced.

## Discussion

Using NPT to guide our process evaluation enabled us to identify the relationship between different stages of the implementation of SHARPS. For example, a lack of a clarity (‘coherence’) about the Peer Navigator role/SHARPS intervention among some staff members inhibited buy-in (‘cognitive participation’), which led to some challenges in making it work (‘collective action’), with tensions manifesting. The SHARPS intervention fitted overall, or perhaps more accurately, in the end. This was partly due to the benefits of the intervention being very visible to all involved, as demonstrated in the qualitative accounts. Yet the challenges regarding ‘fit’ were also intrinsically connected to the benefits: the highly person-centred, flexible Peer Navigator role disrupted organisational structures and practices. We recognise the pressures experienced by frontline staff in this sector with high levels of burnout and staff turnover reported, partly in response to client experiences of trauma^30,31,37^. The Peer Navigators also likely received much more support from a range of people than those in other roles. Therefore, we understand why an ostensibly more liberated role, unencumbered by bureaucratic processes and associated paperwork, with additional support and supervision, could evoke some uncomfortable feelings amongst other staff members in the services involved, although similar roles exist elsewhere. Another key dimension to the intervention’s perceived value was the Peer Navigators’ lived experience, particularly around the compassion they brought to the role. However, as described in the interviews, these experiences were not universally welcomed, at least at first. Recent ‘state of the art’ reviews have highlighted that peers’ experience of stigmatising attitudes from others, poorly defined roles, and poor remuneration (alongside other challenges) are commonplace^12,38^. Some of the Peer Navigators did encounter challenges in negotiating their experiences of abstinence and its ideology whilst working within a harm reduction ethos. They valued the range of formal and informal supports they could draw on in the role. Finally, we considered SHARPS to be a *particularly* complex, complex intervention. For example, in light of the broad geographic range, the hosting of Peer Navigators across six settings and in different not-for-profit organisations. NPT encouraged us to carefully consider each context, including the extent to which there was a harm reduction ethos, and act responsively when required. Such challenges have been considered in detail within our next stage RCT^39^.

Overall, NPT was a useful framework for our process evaluation. We concur with others^16^ that NPT’s focus on the operational actors somewhat neglects patient views/experiences, and in our own data we found that the core constructs did not relate so well to the intervention participant interviews. For example, ‘reflexive monitoring’ seemed more applicable to interviews with the Peer Navigators and staff, than the intervention participants interviews. However, had intervention participants been consulted through a ‘user feedback’ approach, then reflexive monitoring may have felt more applicable. Indeed, the focus on actions of ‘professionals’ rather than ‘end users’ is an identified criticism of NPT^40^. Moreover, like others^41^, sometimes we found the concepts confusing, partly due to the terminology being rather academic, and on occasion were required to remind ourselves of definitions. The confusion was partly caused by the connectedness of the concepts where we were sometimes unsure which of the constructs the data corresponded best with; a challenge which others have also encountered^42–44^. Lastly, we found, again as others have^16,45^ that some of the themes or data sources were not relevant to NPT or did not fit closely with it. For example, some of the reflections shared by the Peer Navigators on their own ‘journeys’ were relevant to NPT and to assessing intervention ‘fit’ in some regards (e.g., reflections on tensions between harm reduction and abstinence), while others were much more personal (e.g., reflections on careers and next steps) and were therefore less relevant to the key aim of assessing intervention feasibility, accessibility and acceptability, and therefore to NPT. They were still important and meaningful insights and have been reported in an associated paper^46^. Regarding data sources ‘reflexive monitoring’ did not seem very applicable to the participant interviews. As mentioned, others have had similar experiences when engaging with NPT and we recognise that NPT is intended to be flexible and not a ‘conceptual straitjacket’^18^. However, we nevertheless welcome the publication of a coding manual^47^ which we view as likely optimising the usage of NPT, for example by providing clarification for researchers about where it could be used, while also confirming where NPT is indeed less suited or not applicable.

As discussed in some detail here, there were some implementation challenges where ‘fit’ was sometimes questioned. In this context, it may have been easy to lose sight of the study aims. Ultimately, NPT’s clear focus on ‘real-world’ application^48^ ensured we focused on identifying and responding to ‘fit’ throughout, asking questions such as: does the intervention ‘fit’? Can we make it fit? How do we make it fit ‘better’? Answering these questions appropriately was central to understanding how the intervention can be enhanced to best respond to the complex and diverse needs of the cohort.

The overall conclusion of the study was that the SHARPS intervention was feasible and acceptable to intervention participants, staff, and the Peer Navigators^6,14^. Through NPT we identified challenges and areas to improve to enhance the intervention. NPT was a useful underpinning theoretical framework for the SHARPS study process evaluation. Its dynamic emphasis enabled our team both to optimise the fit of the intervention during its implementation as far as possible, as well as understand and assess the feasibility and acceptability of the intervention for this population group. The findings presented in this paper along with the findings from the wider feasibility study^6,14^ indicate that the clinical effectiveness of an optimised intervention should be tested via an RCT.

To our knowledge, this is the first application of NPT to an intervention/study involving individuals experiencing these challenges. This demonstrates the utility and breadth of NPT—for instance, in being successfully applied to a health-oriented intervention by non-clinicians (peers), in non-healthcare settings (i.e., third sector, homelessness services). As such, and in sharing our learning and reflections, as well as the first-hand experiences of those directly involved and impacted, we believe this paper offers an important contribution to literature on harm reduction-based behavioural interventions for people experiencing problem substance use (substance use disorders), and homelessness, as well as literature on health and healthcare interventions in this area.

## Supplementary information

Reporting Summary

Supplementary file 1

## Data Availability

Due to the sample size and known geographical locations, there is a risk that individuals may be identified if the datasets were made available. As the interview transcripts contain a considerable amount of contextual data, it may be possible to identify participants, including the members of staff who were interviewed. This study involved important partnerships with a range of organisations with whom the study team have developed trusting working relationships, with the expectation that any arising sensitivities would be carefully considered. For these reasons, the qualitative data sets are not available for sharing. We have applied this policy across all publications, including the monograph.
