# Supplementary material for: Applying Normalisation Process Theory to a peer-delivered complex health intervention for people experiencing homelessness and problem substance use
Source: Commun Med (Lond). 2025 Jan 10;5:13. doi: 10.1038/s43856-024-00721-6 (PMC11724100; doi:10.1038/s43856-024-00721-6)
Supplement: Supplementary file 1 — Reporting Summary [file 43856_2024_721_MOESM1_ESM.pdf]

nature portfolio

Corresponding author(s): *Double-blind peer review submissions: write DBPR and your manuscript number here instead of author names.*

Last updated by author(s): *YYYY-MM-DD*

Reporting Summary

Nature Portfolio wishes to improve the reproducibility of the work that we publish. This form provides structure for consistency and transparency in reporting. For further information on Nature Portfolio policies, see our Editorial Policies and the Editorial Policy Checklist.

Statistics

For all statistical analyses, confirm that the following items are present in the figure legend, table legend, main text, or Methods section.

n/a	Confirmed	
☒	☐	The exact sample size (*n*) for each experimental group/condition, given as a discrete number and unit of measurement
☒	☐	A statement on whether measurements were taken from distinct samples or whether the same sample was measured repeatedly
☒	☐	The statistical test(s) used AND whether they are one- or two-sided<br>*Only common tests should be described solely by name; describe more complex techniques in the Methods section.*
☒	☐	A description of all covariates tested
☒	☐	A description of any assumptions or corrections, such as tests of normality and adjustment for multiple comparisons
☒	☐	A full description of the statistical parameters including central tendency (e.g. means) or other basic estimates (e.g. regression coefficient) AND variation (e.g. standard deviation) or associated estimates of uncertainty (e.g. confidence intervals)
☒	☐	For null hypothesis testing, the test statistic (e.g. *F*, *t*, *r*) with confidence intervals, effect sizes, degrees of freedom and *P* value noted<br>*Give P values as exact values whenever suitable.*
☒	☐	For Bayesian analysis, information on the choice of priors and Markov chain Monte Carlo settings
☒	☐	For hierarchical and complex designs, identification of the appropriate level for tests and full reporting of outcomes
☒	☐	Estimates of effect sizes (e.g. Cohen's *d*, Pearson's *r*), indicating how they were calculated

Our web collection on statistics for biologists contains articles on many of the points above.

Software and code

Policy information about availability of computer code

Data collection	*Provide a description of all commercial, open source and custom code used to collect the data in this study, specifying the version used OR state that no software was used.*
Data analysis	NVivo version 12

For manuscripts utilizing custom algorithms or software that are central to the research but not yet described in published literature, software must be made available to editors and reviewers. We strongly encourage code deposition in a community repository (e.g. GitHub). See the Nature Portfolio guidelines for submitting code & software for further information.

Data

Policy information about availability of data

All manuscripts must include a data availability statement. This statement should provide the following information, where applicable:

- Accession codes, unique identifiers, or web links for publicly available datasets
- A description of any restrictions on data availability
- For clinical datasets or third party data, please ensure that the statement adheres to our policy

Due to the sample size and known geographical locations, there is a risk that individuals may be identified if the datasets were made available. As the interview transcripts contain a considerable amount of contextual data, it may be possible to identify participants, including the members of staff who were interviewed. This study involved important partnerships with a range of organisations with whom the study team have developed trusting working relationships, with the expectation

that any arising sensitivities would be carefully
considered. For these reasons, the qualitative data sets are not available for sharing. We have applied this policy across all publications, including the monograph.

Human research participants

Policy information about studies involving human research participants and Sex and Gender in Research.

Reporting on sex and gender	Participant gender is not reported in the paper.
Population characteristics	No population characteristics are included in this paper as they are reported in related papers.
Recruitment	Recruitment details are not included in this paper as they are reported in related papers.
Ethics oversight	Full ethical approval for the study was sought and obtained. The University of Stirling's NHS, Invasive and Clinical Research (NICR) ethics committee (NICR 17/18 Paper 2018) provided ethical approval for this study in April 2018, and The Ethics Subgroup of the Research Coordinating Council of The Salvation Army (TSA) in June 2018 (no reference number provided). In response to necessary protocol amendments, four subsequent submissions to these committees were made, all of which were approved.

Note that full information on the approval of the study protocol must also be provided in the manuscript.

Field-specific reporting

Please select the one below that is the best fit for your research. If you are not sure, read the appropriate sections before making your selection.

☐ Life sciences ☒ Behavioural & social sciences ☐ Ecological, evolutionary & environmental sciences

For a reference copy of the document with all sections, see nature.com/documents/nr-reporting-summary-flat.pdf

Behavioural & social sciences study design

All studies must disclose on these points even when the disclosure is negative.

Study description	Mixed methods study but only qualitative data are reported in this paper.
Research sample	Participants who were receiving the SHARPS intervention (n=24/10); staff working in intervention sites (n=12) and Peer Navigators (n=4)
Sampling strategy	Participants all sampled using purposive sampling. No calculations required as qualitative data.
Data collection	Data collected using semi-structured interviews either by phone or face-to-face by academic researcher or peer researcher.
Timing	2018-2020.
Data exclusions	None
Non-participation	None
Randomization	N/A

Reporting for specific materials, systems and methods

We require information from authors about some types of materials, experimental systems and methods used in many studies. Here, indicate whether each material, system or method listed is relevant to your study. If you are not sure if a list item applies to your research, read the appropriate section before selecting a response.

Materials & experimental systems

n/a	Involved in the study
☒	☐ Antibodies
☒	☐ Eukaryotic cell lines
☒	☐ Palaeontology and archaeology
☒	☐ Animals and other organisms
☒	☐ Clinical data
☒	☐ Dual use research of concern

Methods

n/a	Involved in the study
☒	☐ ChIP-seq
☒	☐ Flow cytometry
☒	☐ MRI-based neuroimaging
