# Supplementary material for: Applying Normalisation Process Theory to a peer-delivered complex health intervention for people experiencing homelessness and problem substance use
Source: Commun Med (Lond). 2025 Jan 10;5:13. doi: 10.1038/s43856-024-00721-6 (PMC11724100; doi:10.1038/s43856-024-00721-6)
Supplement: Supplementary file 2 — Supplementary file 1 [file 43856_2024_721_MOESM2_ESM.docx]

**Supplementary file 1. Interview topic guides**

**Peer Navigators**

Interview 1 (pre-intervention)

- First week- how has it been? What's gone well/less well/ok? First impressions- the study, the team members that you've met, the Salvation Army, the stakeholder organisations you’ve met so far, the academic subject area (problem substance use and homelessness mainly), the university.
- Experiences of seminar and SDF event- atmosphere at these, interactions, topics covered- overall feeling and reflections, any surprises, any challenges, anything a bit tricky?
- Thoughts on the study overall- study background, approach, aims? Do you see any challenges or issues- if so, what are they?
- Intervention/manual development- how did you find the day overall? What are the key elements we need to include/think about in your view? Do we have any challenges associated with manualising? If 'yes', what are they? Anything else?
- Peer Navigator role in the study- what do you see your role as being? Thoughts on this? Aims and any concerns? Comprehensive induction and training planned, and it's early days- but anything that sticks out at this stage that’s missing or not enough that would support you in your role or help your learning/development? Anything that you want to know more about, or do that would help you with your work?
- Any questions or comments? Anything else?

Interview 2 (mid-intervention)

- Experiences in post so far
- Recruitment of participants – any challenges?
- Health checks and outcome measures – how are these going?
- Training, support and supervision – is there anything you need?
- Fidelity – are you doing what is expected as part of the intervention? Are you being asked to do things outwith your role?
- Acceptability - is the intervention/Peer Navigator acceptable to other staff/service users?
- Local context- accessibility of services, any barriers?
- Staff relationships and dynamic – any challenges?
- Suggestions for ways to improve intervention
- Any other challenges?

Additional Peer Navigator interview

- General discussion
- Experiences of retaining participants – working day to day with caseload
- Experiences of participant monies fund – usage, issues, access etc.
- Paired working – views on working with Peer Navigator in service, what would be ideal set up etc.
- Anything else wishing to raise, discuss, ask.

Final interview

- Reflection on intervention/post overall – things enjoyed, things that have been ok, challenges etc.
- Recruitment and retention of participants – experiences, challenges, suggestions for improvements
- Experience of conducting health checks and outcome measures, including change in planned approach. How long on average does it take to arrange the measures (taking account of texts, calls often repeated)?
- Training, support and supervision – anything you wanted to receive? Was support appropriate and sufficient? Were there things we should have told you before you started that we didn’t? Support: WhatsApp group, support from Jason, supervision from Adam, support from Service Managers and study team.
- How about preparing the settings? How was that? What could or should we have done to prepare them better, if anything?
- Fidelity – do you think the intervention/your role went as expected? Were you expected to do things beyond your role? Do you think you delivered the intervention the same as/similarly to the other three Peer Navigators? Why do you think this? Please expand. Is this an intervention that should be delivered exactly the same by each Peer Navigator? Risks of not? Benefits of not?
- Acceptability – is the intervention/Peer Navigator acceptable to other staff? How about to the service users?
- Support for participants – can you give some examples of what you did to support participants? To show breadth of what you do (and your hard work!).
- How well did the practical support monies work out? Any changes we should do here?
- Experience of being part of a research study – hard to disentangle from role of course, but the requirements of being connected to a study e.g. interviews, arranging and supporting the measures, filling in various logs
- If we were doing study again, would you make any changes? If so, what would they be?
- And relatedly, overall do you think the study is feasible to be rolled out further? To help answer, what worked really well? What worked less well? Operationally how could it have been improved upon?
- Thinking about a potential next stage study, do you see this as being able to be applied in other settings? Within the area and beyond e.g. social work, hospitals.
- Based on your experience and related to above, what would your thoughts/feelings be about randomising this intervention?
- Next steps – what are you doing next and why? Or what are you wanting to do next and why? Decision making underlying this e.g. wanted similar role, wanted different role
- Would you recommend the role to someone in your position (your position 18 months ago)?
- Anything else? Questions, comments, feedback

**Staff**

**Staff – intervention settings**

- Experiences of the intervention within the service
- Fit of the intervention/Peer Navigator role within the service- sense of how this working/not working including relationships with existing staff team
- Related to above – fit with existing roles within the service or within your organisation as a whole, similarities and differences between roles?
- How different Peer Navigators are working- thoughts, reflections
- Peer Navigator skills and training
- Recruitment of intervention participants
- Health checks and outcome measures
- Training, support and supervision
- Fidelity to the intervention – are the Peer Navigators doing what is expected of them?
- Acceptability – is the intervention/Peer Navigator acceptable to other staff/service users?
- Suggestions for ways to improve intervention
- Challenges
- Do you see this as being able to be applied in other settings? Within the area and beyond e.g. social work, hospitals.
- Related to above, do you think this intervention could be randomised? If so, what would your thoughts/feelings on randomisation be?

**Peer Research Wave One – Sample. These were tailored to each Peer Navigator.**

**NOTES**

Confidentiality – will only be broken if person discloses harm to themselves or others

Introduce yourself – name, Peer Researcher from SDF, today we’d like to ask you a few questions about how you’ve found working with [Peer Navigator name], should take 20-40 minutes.

Recording – interviews are being recorded to make it easier for us, so we don’t have to take notes. No one else will hear the recording except the person typing it up and the research team.

1. Read information sheet (or pass to person to read themselves).
2. Ask if they have any questions
3. Compete 2 copies of the consent form – one for them to keep and one for the researchers. You sign both copies as ‘researcher’. Make sure they tick each box on both copies.
4. Start recording after completing consent form – check that they are happy to start the interview
5. At the end of the interview, stop recording and then give debrief sheet and £10 voucher
6. Afterwards, debrief with [researcher/User Involvement Officer] and give [researcher] recording and consent form.

Below each question is a list of possible prompts, but you can also ask other prompts that you think are relevant, for example: “can you tell me a bit more about that?’; ‘can you say a bit more about that?’ ‘can you explain what you mean by that’?

***Begin the interview – start the recording***

- **What do you think about the accommodation and/or support you are receiving here at [service name]?**
  - What do you like/dislike?
  - Any suggestions for improvements
- **Have you made use of any other (similar) services before this?**
  - If yes, how does this service compare?
  - Experience of peer support?
- **How/where did you hear about/meet Peer Navigator?**

How did you find recruitment to the study e.g. being provided with info/consent etc?

- - How long did it take for you to start working with Peer Navigator?
  - How easy was it to start working with Peer Navigator?
  - Would you change anything about the process? If yes, what/why?
- **Can you tell me a bit about what have you been doing with Peer Navigator?**

What kind of things have you been doing? What does he help you with?

- - How have you found the support provided by Peer Navigator? Does it happen on time/when it’s supposed to?
  - Have you been to any appointments? Have they helped you access support/services?
  - Have they provided emotional support – do you talk to Peer Navigator about what’s going on for you? Are they supportive?
- **How have you found working with Peer Navigator?**
  - How useful has it been?
  - What did you get out of it?
  - Can you tell me a bit about you relationship with Peer Navigator (e.g. dynamic, pace, being able to be open/honest, building rapport)
  - Likes/dislikes/suggestions?
  - How have you found the approach of the study?
- **Have you made any changes to your life/behaviour since working with Peer Navigator?**
  - For example, accessed health services, housing, harm reduction etc?
  - If not, do you think you will in the future? Do you feel encouraged/supported to do so?
- **Have you met with [researcher] and have you completed some questionnaires about your health?**
  - If yes, how did it go? Did you feel comfortable?
  - If not, why not? What might help you to feel comfortable/ready?
  - How do you feel doing this with a researcher rather than with Peer Navigator?
- **How does working with Peer Navigator compare to other support/services you have received?**
  - What’s similar/different?
  - Why do you think that’s different?

- **Would you recommend the Peer Navigator service/intervention to someone in your position?**
  - Why/why not?
- **Is there anything you would like to say about how Peer Navigator works here in this service?**
- **Do you have anything further to add that has not been covered?**

**Peer Research Wave Two – Sample. These were tailored to each Peer Navigator.**

**NOTES**

Confidentiality – will only be broken if person discloses harm to themselves or others

Introduce yourself – name, Peer Researcher from SDF, today we’d like to ask you a few questions about how you’ve found working with Peer Navigator, should take 20-40 minutes.

Recording – interviews are being recorded to make it easier for us, so we don’t have to take notes. No one else will hear the recording except the person typing it up and the research team.

- Thank them for their participation in the interview last time and say how helpful it was.
- Explain that some questions are similar to last time and some are new. The aim of doing this a second time is to see how things are now (e.g. if anything has changed) and generally capture any new information now that they have been working with Peer Navigator for a bit longer. This is really important for us.
- Read through information sheet (or pass to person to read themselves).
- Ask if they have any questions
- Compete 2 copies of the consent form – one for them to keep and one for the researchers. You sign both copies as ‘researcher’. Make sure they tick each box on both copies.
- Start recording after completing consent form – check that they are happy to start the interview
- At the end of the interview, stop recording and then give debrief sheet and £10 voucher
- Afterwards, debrief with [researcher/User Involvement Officer] and give [researcher] recorder and consent form.

Below each question is a list of possible prompts, but you can also ask other prompts that you think are relevant, for example: ‘can you tell me a bit more about that?’; ‘can you say a bit more about that?’ ‘can you explain what you mean by that’?

***Begin the interview – start the recording***

- **So that the study team can match this interview to the last one, please can you give your name? First name is fine and it will be replaced with your participant ID code (you will not be able to identified).Thank you.**
- **What do you think about the accommodation and/or support you are receiving here at [service]?** (NOTE for Researcher – the participant may not be using this service or may not be using it regularly)
  - What do you like/dislike?
  - Any suggestions for improvements
- **Can you tell me a bit about what have you been doing with [Peer Navigator], since you last spoke to a Peer Researcher (which was in February)?**
- What kind of things have you been doing? What does he help you with?
  - How have you found the support provided by Peer Navigator? Does it happen on time/when it’s supposed to?
  - Have you been to any appointments? Has he helped you access support/services?
  - Has he provided emotional support – do you talk to Peer Navigator about what’s going on for you? Is he supportive?
- **How have you found working with Peer Navigator since February?**
  - How useful has it been?
  - What did you get out of it?
  - Can you tell me a bit about you relationship with Peer Navigator (e.g. dynamic, pace, being able to be open/honest, building rapport)
  - Likes/dislikes/suggestions?
  - How have you found the approach of the study?
- **Have you made any changes to your life/behaviour since February that you would say are linked to working with Peer Navigator?**
- For example, accessed health services, housing, harm reduction etc?
- If not, do you think you will in the future? Do you feel encouraged/supported to do so?
- **Did you meet with [study researcher] before to complete some questionnaires about your health?**

**If no –**

- If you remember, why not?
- Is there anything that the study team could have done differently to make you feel more comfortable about doing them?

**If yes –**

- How did it go last time?
  - How are you feeling about doing them again with [researcher]?
  - If not comfortable/ready, what might help you to feel comfortable/ready?
  - How do you think your answers will compare to last time?
- **How does working with [Peer Navigator] compare to other support/services you have received?**
  - What’s similar/different?
  - Why do you think that’s different?

- **Would you recommend the Peer Navigator service/intervention to someone in your position?**
  - Why/why not?
- **Is there anything you would like to say about how [Peer Navigator] works here in this service?**
- **How do you feel about finishing up work with [Peer Navigator] (when his post finishes)?** (NOTE for Researcher: this conversation between participant and [Peer Navigator] may not have happened yet so ‘don’t know’ is fine for answers to these)
- What do you plan on doing next?
- What support has [Peer Navigator] put in place for you?
- **In these last couple of questions I’d like to ask you for your thoughts please on the study and a potential follow up study to this one. Please bear with me while I tell you a bit more.**

**As you know, this is a research study – it is a pilot study and the idea is that a larger, follow-on study may happen if the findings from this one are promising.**

**This study would be a Randomised Control Trial (also known as an RCT). You might have heard of them before – they are used most often in medical research.**

**In an RCT, a number of similar people are randomly assigned to 2 (or more) groups to test a specific drug, treatment or other intervention.**

**In this case, it would be testing the Peer Navigator intervention.**

**It would mean participants would all agree to be part of a study, but some people would get a Peer Navigator and others would not (they would instead receive support from other members of staff, as they would normally). How people were getting on would then be compared to see if the Peer Navigator role/intervention is making a real difference to people’s lives.**

- What do you think about this idea?
- How you think others in a similar position to yourself would find being randomised?
- Would you have any issues with it or concerns about it?
- **Another part of doing an RCT is that the study team would look to link participants data.**

**Only a small number of individuals from the study team would have access to the data and would analyse it.**

**The team would analyse and link different kinds of data – for example, GP data, hospital admission data, prison admission data – to determine if someone is going to the doctors or a hospital less often, or is going to prison less often, because they are now working with a Peer Navigator and that is going well (these are some examples). This is a way of determining if the Peer Navigator role/intervention is making a real difference to people’s lives.**

- What do you think about this idea?
- How do you think others in a similar position to yourself would be about having their data analysed and linked?
- Would you have any issues or concerns about it?
- **Do you have anything further to add that has not been covered?**

Thank for time and give debrief sheet and voucher.
